# Detection of *NPM1 *exon 12 mutations and *FLT3 *– internal tandem duplications by high resolution melting analysis in normal karyotype acute myeloid leukemia

**DOI:** 10.1186/1756-8722-1-10

**Published:** 2008-07-29

**Authors:** Angela YC Tan, David A Westerman, Dennis A Carney, John F Seymour, Surender Juneja, Alexander Dobrovic

**Affiliations:** 1 Department of Pathology, Peter MacCallum Cancer Centre, Melbourne, Australia; 2 Department of Haematology and Medical Oncology, Peter MacCallum Cancer Centre, Melbourne, Australia; 3 Department of Pathology, University of Melbourne, Parkville, Australia; 4 Royal Melbourne Hospital, Parkville, Australia

## Abstract

**Background:**

Molecular characterisation of normal karyotype acute myeloid leukemia (NK-AML) allows prognostic stratification and potentially can alter treatment choices and pathways. Approximately 45–60% of patients with NK-AML carry *NPM1 *gene mutations and are associated with a favourable clinical outcome when *FLT3*-internal tandem duplications (ITD) are absent. High resolution melting (HRM) is a novel screening method that enables rapid identification of mutation positive DNA samples.

**Results:**

We developed HRM assays to detect *NPM1 *mutations and *FLT3*-ITD and tested diagnostic samples from 44 NK-AML patients. Eight were *NPM1 *mutation positive only, 4 were both *NPM1 *mutation and *FLT3*-ITD positive and 4 were *FLT3*-ITD positive only. A novel point mutation Y572C (c.1715A>G) in exon 14 of *FLT3 *was also detected. In the group with *de novo *NK-AML, 40% (12/29) were *NPM1 *mutation positive whereas *NPM1 *mutations were observed in 20% (3/15) of secondary NK-AML cases. Sequencing was performed and demonstrated 100% concordance with the HRM results.

**Conclusion:**

HRM is a rapid and efficient method of screening NK-AML samples for both novel and known *NPM1 *and *FLT3 *mutations. *NPM1 *mutations can be observed in both primary and secondary NK-AML cases.

## Background

Acute myeloid leukemia with a normal karyotype (NK-AML) is considered to have an intermediate prognostic risk with 5 year disease free survival (DFS) ranging between 24–42% [1,2]. However, there is marked variability in outcome suggesting significant biological and molecular heterogeneity within this group of AML [3].

In 2005, Falini *et al*. described a set of common mutations within the final exon of the *NPM1 *gene in primary NK-AML patients, which alter the N-terminal domain nuclear localisation signal leading to abnormal cytoplasmic accumulation of the NPM1 phosphoprotein [4]. While the precise functional effect of the *NPM1 *mutation is incompletely understood, several groups confirmed that NK-AML patients have a high incidence of *NPM1 *exon 12 mutations (~24% – 60%) [5-9]. Mutations in *NPM1 *are the most frequent genetic change known in patients with NK-AML and a number of studies have shown that *NPM1 *mutation positive patients have a better prognosis with longer event-free and overall survival (OS) [10].

Schnittger *et al. *demonstrated that the favourable prognostic implications of *NPM1 *mutation status are overridden in *FLT3*-ITD positive cases which have a uniformly poor prognosis [7]. These findings demonstrate the need to screen patients for mutations in *FLT3*-ITD alongside *NPM1 *[10]. However, such a molecular screening program can be demanding on the resources of a diagnostic laboratory. Therefore, in this study we assessed the use of high resolution melting (HRM) analysis as a rapid method to screen NK-AML patient samples for the critical molecular changes in *NPM1 *and *FLT3*.

## Results and Discussion

In this study, we developed HRM assays allowing rapid assessment of the mutation status of *NPM1 *and the presence of the *FLT3*-ITD in the same run. In HRM, the PCR product is subjected to melting in the presence of a dye that only fluoresces when bound to double stranded DNA [11]. As melting is sequence dependent, monitoring the precise melting behaviour by observing the change in fluorescence allows the detection of variant sequences. In addition, sequence variants in the DNA such as mutations give rise to heteroduplexes that form earlier melting products allowing ready detection of mutations even at comparatively low concentrations.

**Figure 1:**
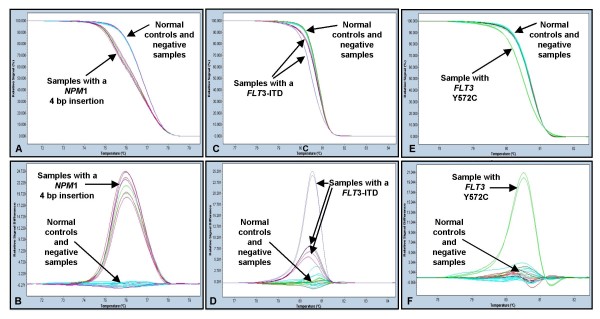
**Detection of NPM1 mutations and FLT3-ITD using high resolution melting analysis.** (A) The melt curve of NPM1 exon 12 and (B) The difference plot of NPM1 exon 12. Six patient samples are shown in comparison to five normal controls.  Four patients (#6, #12, #14 and #38) are NPM1 mutation positive and two patients (#33 and #43) are NPM1 mutation negative. (C) The melt curve of FLT3 exon 14 and (D) The difference plot of FLT3 exon 14 - Six patient samples are shown in comparison to five normal controls.  Three patients (#6, #33 and #43) are FLT3-ITD positive and three patients (#12, #14 and #48) are FLT3-ITD negative. (E) The melt curve of FLT3 exon 14 and (F) The difference plot of FLT3 exon 14 - Eight patient samples are shown in comparison to five normal controls.  One patient (#19) is positive for FLT3 Y572C and seven patients (#4, #5, #10, #24, #25, #26 and #30) are FLT3 mutation negative.  All samples are shown in duplicate.

Samples from 44 patients with NK-AML were analysed. The median age of the patients was 62 years (range 18–89 years) and 27 (61%) patients were male. Twenty nine (66%) had *de novo *AML and 15 (34%) had secondary AML. Sixteen patients generated an abnormal melting profile in one of the two tested amplicons, 8 were *NPM1 *mutation positive only, 4 were *NPM1 *positive and *FLT3*-ITD positive and 4 were *FLT3*-ITD positive only (Figure 1.).

Sequencing confirmed all the HRM detected mutations and did not reveal any further mutations, indicating that HRM was capable of detecting mutations with 100% sensitivity in this cohort.

All the *NPM1 *mutations detected involved one of two 4 base insertions that altered the tryptophan at amino acid position 288 and the *FLT3*-ITD ranged from 33–102 bases (Table 1). These mutations were similar to those previously described [4,12,13]. All 12 *NPM1 *mutation positive patients were also positive by immunohistochemistry (IHC) on bone marrow trephine sections, showing typical cytoplasmic localisation (data not shown).

**Table 1:** Patient demographics and list of *NPM1 *and *FLT3*-ITD mutations detected

#	Age	Sex*	FAB	Prior Disease†	HRM – NPM1‡	Seq – NPM1§	HRM – FLT3- ITD	Seq-FLT3-ITD|
1	64	M	M1	Nil	Normal	Neg	Normal	Neg
2	63	M	M6	MDS	Normal	Neg	Normal	Neg
3	36	M	M2	Nil	Normal	Neg	Normal	Neg
4	69	M	basophilic leukemia	RAEB-T	Normal	Neg	Normal	Neg
5	68	M	M2	Nil	Normal	Neg	Normal	Neg
6	81	M	M5	Nil	Aberrant	860_863*dup*TCTG	Aberrant	1754_1798*dup*
7	19	M	M2	Nil	Normal	Neg	Normal	Neg
8	68	M	M6	Nil	Normal	Neg	Normal	Neg
9	72	M	M4	CMML	Normal	Neg	Normal	Neg
10	58	F	M2	Nil	Aberrant	860_863*dup*TCTG	Normal	Neg
11	66	F	M4/5	MDS transformed	Aberrant	860_863*dup*TCTG	Normal	Neg
12	51	F	M4	Nil	Aberrant	860_863*dup*TCTG	Normal	Neg
13	42	F	M2	MDS/pelvic chloroma	Normal	Neg	Normal	Neg
14	53	M	M4	Nil	Aberrant	860_863*dup*TCTG	Normal	Neg
15	59	F	M1	Nil	Aberrant	860_863*dup*TCTG	Aberrant	1811_1837*dup *1838_1867*ins*
16	69	M	M1	Nil	Normal	Neg	Normal	Neg
17	52	M	M1	MDS	Normal	Neg	Normal	Neg
18	74	M	M1	Nil	Normal	Neg	Normal	Neg
19	66	M	M0	ca prostate	Aberrant	860_863dupTCTG	Aberrant	1715A>G
20	52	M	M1	MDS	Normal	Neg	Normal	Neg
21	75	M	M1	MDS	Normal	Neg	Normal	Neg
22	56	M	M0	NHL on TX	Normal	Neg	Normal	Neg
23	18	F	M1	Nil	Normal	Neg	Normal	Neg
24	73	M	M4	Nil	Normal	Neg	Normal	Neg
25	56	F	M1	therapy related	Normal	Neg	Normal	Neg
26	53	F	M4	Nil	Aberrant	860_863*dup*TCTG	Normal	Neg
27	64	M	M0	MDS	Normal	Neg	Normal	Neg
28	63	F	M5a	Nil	Aberrant	861_864*ins*CTGC	Normal	Neg
29	71	F	M5b	MDS transformed	Aberrant	860_863*dup*TCTG	Aberrant	1754_1789*dup*
30	89	F	M6	Nil	Normal	Neg	Normal	Neg
31	28	F	M4	Nil	Normal	Neg	Normal	Neg
32	69	F	basophilic leukemia	Nil	Normal	Neg	Normal	Neg
33	36	F	M5b	Nil	Normal	Neg	Aberrant	1783_1812*dup*
34	54	M	M1	Nil	Normal	Neg	Normal	Neg
35	52	M	M1	Nil	Normal	Neg	Normal	Neg
36	82	M	M4	Nil	Normal	Neg	Aberrant	1786G>C 1787_1818*dup*
37	68	M	M5b	CMML	Normal	Neg	Normal	Neg
38	61	F	M4	Nil	Aberrant	860_863*dup*TCTG	Normal	Neg
39	49	M	M4	Nil	Normal	Neg	Aberrant	ins ? bp ¶|
40	83	F	M1	Nil	Aberrant	860_863*dup*TCTG	Normal	Neg
41	72	F	M4	CMML	Normal	Neg	Normal	Neg
42	57	M	M4	Nil	Normal	Neg	Normal	Neg
43	42	M	M1	Nil	Normal	Neg	Aberrant	1741_1831*dup *1832_1842*ins*
44	49	M	M6	Nil	Normal	Neg	Normal	Neg

† MDS = myelodysplastic syndrome; RAEB-T = refractory anemia with excess of blasts in transformation; CMML = chronic myelomonocytic leukemia; Ca prostate = prostate cancer; NHL = Non-Hodgkin lymphoma.

‡Normal = normal melt profile, Aberrant = abnormal melt profile.

§Neg = no mutation detected in the sequence; numbering according to *NPM1 *reference sequence NM_002520.5

| Numbering according to *FLT3 *reference sequence NM_004119.2 (5'UTR not included)

¶ The size of the internal tandem duplication could not be determined due to the low levels of mutant peaks in the sequence,

The incidence of *NPM1 *mutations in the *de novo *AML cases was 40% (12/29), consistent with the incidence reported in previous studies [5-9]. Interestingly, 3/15 of the secondary AML cases were *NPM1 *mutation positive which contrasts with an earlier study, where cytoplasmic localisation of NPM indicative of *NPM1 *mutations was not seen in 135 secondary AML samples by IHC [4].

A novel point mutation Y572C in exon 14 of *FLT3 *was also detected. This tyrosine residue within the juxtamembrane domain of *FLT3 *has been shown to be phosphorylated *in vivo *[14] and could be included in the newly described class of *FLT3 *juxtamembrane domain point mutations for which the similar mutation Y591C has been reported [15]. This illustrates the power of HRM to detect novel as well known mutations. The use of HRM to screen for *FLT3*-ITD has been previously reported [16].

HRM is rapidly becoming the most important mutation scanning methodology. It is an in-tube method, meaning that PCR amplification and subsequent analysis are sequentially performed in the one tube or well. This makes it more convenient than other scanning methodologies such as denaturing high-performance liquid chromatography [17]. We used a real-time PCR machine with HRM capability rather than a stand-alone HRM instrument. This facilitates quality control as the success of the amplification can be assessed on the same platform as the melting analysis.

HRM has no real disadvantages in mutation scanning except that extra care needs to be taken in designing PCR reactions to avoid primer dimers and non-specific amplification. Secondly, DNA needs to be prepared in a uniform fashion to avoid variation in salt concentration that will affect the melting. In addition, the exact nature of any mutation cannot be determined without sequencing. Nevertheless, performing HRM as an initial screen for potential mutations significantly reduces the volume of samples requiring sequencing with consequent reduction of cost and labour, and improvements to turn around time.

## Conclusion

HRM is likely to play a major role in clinical applications as it enables rapid detection of defined and novel molecular changes in clinical samples. In this study, the conditions have been optimised to enable screening of normal karyotype AML patients for both *NPM1 *and *FLT3*-ITD in the same run. This has enhanced patient prognostication and clinical decision making regarding therapeutic approaches. The assays are suitable both for individual patient diagnosis and for large scale clinical trials.

## Methods

### Patients and samples

DNA was extracted from archival bone marrow smears from 44 NK-AML patients from 1999–2007 sent to the Pathology Department of The Peter MacCallum Cancer Centre. Normal peripheral blood samples were obtained from 11 healthy volunteers. All samples were collected and were obtained in accordance with the Peter MacCallum Cancer Centre Ethics of Human Research guidelines. DNA was extracted from bone marrow smears using a standard phenol/chloroform extraction technique. DNA was extracted from peripheral blood using the Wizard Genomic DNA Purification Kit (Promega, Madison, WI).

### High resolution melting analysis

The PCR and melting analysis for *NPM1 *and *FLT3 *mutations were all performed on the LightCycler 480 (Roche Diagnostics, Penzberg, Germany) a real-time PCR machine with HRM capability and a 96/384 well capacity. All samples were tested in duplicate. At least 5 different normal controls for each gene were included in each run. Approximately 10 ng of DNA was amplified in a total volume of 10 μL containing 400 nM each of the relevant forward and reverse primer (NPMex12F-TGATGTCTATGAAGTGTTGTGGTTCC, NPMex12R-CTCTGC ATTATAAAAAGGACAGCCAG; or *FLT3*ex14F-TGCAGAACTGCCTATT CCTAACTGA; *FLT3*ex14R-TTCCATAAGCTGTTGCGTTCATCAC, 4 mM (*NPM1*) or 3 mM (*FLT3*) MgCl_2_, and LightCycler 480 High-Resolution Melting Master (Roche Diagnostics). The cycling conditions were the same for both amplicons allowing them to be performed in the one run. The conditions were 95°C (10 min) and a touch down of 10 cycles of 95°C (10 sec), 65°C–55°C (10 sec, 1°C/step), 72°C (30 sec) and a further 45 cycles. The melting program was 95°C (1 min) 45°C (1 min), then 65°C–95°C (5 sec, 1°C/sec). Thirty acquisitions were collected per °C. Upon completion of the run (approximately 2 hours), analysis was performed using the software supplied with the LightCycler 480. The melting curves were normalised and temperature shifted to allow samples to be directly compared. Difference plots were generated by selecting a negative control as the baseline and the fluorescence of all other samples was plotted relative to this sample. Significant differences in fluorescence were indicative of mutations.

### Sequencing

Sequencing was performed on all samples. Approximately 10 ng of DNA was amplified in a total volume of 25 μL containing 200 nM each of M13 tagged primers, 2 mM MgCl_2_, 200 μM each dNTPs, 0.5 units FastStart Taq (Roche Diagnostics) and 1× Buffer. The primers used were the same as stated above except that the M13 sequences 5' TGTAAAACGACGGCCAGT and 5' CAGGAAACAGCTATGACC were tagged to the forward and reverse primers respectively. The cycling conditions were 95°C (10 min) and 45 cycles of 94°C (30 sec), 64°C (30 sec), 72°C (30 sec) and 72°C for 10 min. The products were checked on a 2% ethidium bromide stained agarose gel before sequencing.

## Competing interests

Alex Dobrovic has received honoraria from Roche Diagnostics for speaking about HRM.

## Authors' contributions

AYCT wrote the paper and performed the experiments, AD developed the assay with AYCT, co-wrote the paper and revised the paper in accordance with the reviewers' comments, DAW, DC, and JFS initiated the project, provided the specimens and assisted with writing, SJ provided specimens and performed the immunohistochemical analysis. All authors read and approved the final manuscript.

## Note added in proof

After this manuscript was submitted, another report of *NPM1 *mutations in secondary AML has appeared [18].
